# FGF-23 Levels before and after Renal Transplantation

**DOI:** 10.1155/2009/379082

**Published:** 2009-05-17

**Authors:** Domniki Economidou, Spyros Dovas, Aikaterini Papagianni, Panagiotis Pateinakis, Dimitrios Memmos

**Affiliations:** University Department of Nephrology, Hippokration General Hospital, 54642 Thessaloniki, Greece

## Abstract

Phosphatonin fibroblast growth factor-23 (FGF-23) is involved in phosphate (P) excretion and vitamin D metabolism. Recently, FGF-23 has been suggested to be responsible for the hypophosphatemia and inappropriately low calcitriol levels observed after renal transplantation. We performed a prospective study to investigate FGF-23 levels in patients with end-stage renal disease before and after renal transplantation and their probable association with markers of bone and mineral metabolism. Intact FGF-23 levels were determined before and at 3, 6, and 12 months posttransplantation in 18 renal transplant recipients. Intact parathyroid hormone (iPTH), calcium (Ca), P, 25(OH)VitD, and 1,25(OH)_2_VitD levels were measured at the same time periods. Renal threshold phosphate concentration (TmPO_4_/GFR) was also calculated at 3, 6, and 12 months posttransplantation. The results showed that FGF-23 levels decreased by 89% 3 months posttransplantation (346 ± 146 versus 37 ± 9 pg/mL, *P* < .01) and remained stable throughout the study period. iPTH and P levels also decreased significantly after renal transplantation, while Ca and 1,25(OH)_2_VitD increased. Pretransplantation FGF-23 was significantly correlated with P levels at 3 months posttransplantation (*P* < .005). In conclusion, FGF-23 levels decrease dramatically after successful renal transplantation. Pre-transplantation FGF-23 correlate with P levels 3 months posttransplantation.

## 1. Introduction

Phosphatonin fibroblast growth factor-23 (FGF-23) is involved in phosphate (P) excretion and vitamin D metabolism. FGF-23 induces phosphaturia and inhibits renal 1-a hydroxylase leading to decreased calcitriol synthesis [1]. As a result disorders of FGF-23 excess are characterized by hypophosphatemia with increased renal phosphate wasting and inappropriately low calcitriol levels for the degree of hypophosphatemia [2]. Recently, FGF-23 has been suggested to be responsible for the hypophosphatemia and inappropriately low calcitriol levels observed after renal transplantation [3–6]. Still though, its role in the hypophosphatemia observed after renal transplantation remains largely unknown. The aim of the present prospective study was therefore to investigate FGF-23 levels in patients with end-stage renal disease before and after a successful renal transplantation and their probable association with markers of bone and mineral metabolism.

## 2. Patients and Methods

Eighteen renal transplant recipients (M/F 9/9, mean age 31 ± 11 years) consecutively entered the study. Primary diseases were chronic glomerulonephritis (*n* = 4), vesicureteral reflux (*n* = 4), hereditary/cystic kidney disease (*n* = 3), tubulointerstitial disease (*n* = 1), and unknown etiologies (*n* = 6). Fifteen patients (83%) received a living related and three a deceased donor kidney transplants. Sixteen patients received a first transplant and two a second one. Fourteen patients had been on dialysis before transplantation (median [interquartile range] dialysis duration 16 [11–42] months) and four patients had a pre-emptive transplantation. Maintenance immunosuppression consisted of corticosteroids (100%), a calcineurin inhibitor (cyclosporine *n* = 17, 94% or tacrolimus *n* = 1, 6%) and mycophenolate mofetil (MMF) (*n* = 16, 89%) or everolimus (*n* = 2, 11%). Two doses of 500 mg intravenous methylprednisolone were administered in 11 patients, one on the day before and the other on the day of renal transplantation. In the remaining 7 patients the same dose was administered once, on the day of renal transplantation. A mean dose of 80 ± 40 mg was administered on the first postoperative day. Then methylprednisolone was gradually tapered to a dose of 4–6 mg daily by the end of the first year. Cyclosporine and tacrolimus concentrations were controlled according to standard protocols. MMF dosage was adjusted in case of intolerance. The majority of the patients (*n* = 14, 78%) received induction therapy with basiliximab. All rejection episodes (five in total) were treated successfully with corticosteroid pulse therapy.

Six patients (33%) had a history of parathyroidectomy prior to transplantation. Thirteen patients (83%) were receiving alfacalcidol (One-Alpha, Leo, Princes Risborough, Buckinghamshire, UK) after transplantation, 6 of them in combination with alendronate (Fosamax, Merck & Co., Inc., USA). Alfacalcidol was administered for osteoporosis prophylaxis to all patients except those with hypercalcemia (Serum Ca >10,5 mg/dL) and those with 1,25(OH)_2_VitD levels greater than 54 pg/mL. Six patients (33%) were receiving calcium salts.

Intact FGF-23 levels were determined by enzyme-linked immunosorbent assay (ELISA) (Immutopics, San Clemente, CA, USA) before and 3, 6, and 12 months posttransplantation. The intra- and interassay precision was 4.4% and 6.1% coefficient of variation. Intact parathyroid hormone (iPTH), calcium (Ca), P, 25(OH)VitD, and 1,25(OH)_2_VitD levels were measured at the same time periods. iPTH was determined by IRMA (IMMUNOTECH, Marseilles, France), and 25(OH)VitD, and 1,25(OH)_2_VitD were determined by RIA (BioSource S.A., Brussels, Belgium). Serum creatinine (S_cr_), Ca, P, and urine creatinine, and P were measured by standard techniques using an automated analyzer (Olympus AV 560, Hamburg, Germany). The renal threshold of P concentration standardized to glomerular filtration rate (TmPO_4_/GFR) was used as a measure of renal P handling [7]. All patients had satisfactory renal function at month 3 posttransplantation and remained stable until the end of the study (S_cr_: 1.3 ± 0.2 mg/dL). Glomerular filtration rate (GFR) was estimated using MDRD formula and measured with ^51^Cr-EDTA between 6 and 12 months posttransplantation when S_cr_ was stable. Mean eGFR(MDRD) was 62.06 ± 15.76 mL/min/1.73 m^2^, and mean ^51^Cr-EDTA GFR was 50 ± 18 mL/min/1.73 m^2^.

Statistical analysis was performed using SPSS 15.0 for Windows (SPSS Inc., Chicago, Illinois). Data are expressed as mean ± SD (parametric parameters) or median with interquartile range (non-parametric parameters), as appropriate. Differences between study periods in different parameters were analyzed using paired *t*-test. Comparisons between groups were analyzed using unpaired *t*-test. Correlations between different parameters were analyzed by Spearman's rank correlation. Two-sided *P* < .05 was considered statistically significant.

## 3. Results

**Figure 1:**
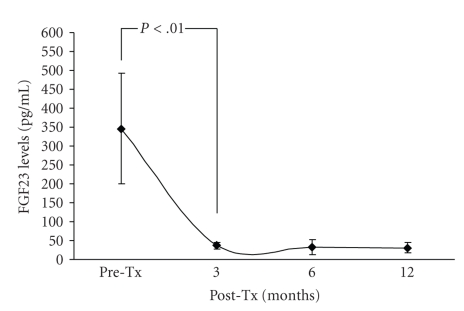
FGF-23 levels before and after successful renal transplantation (Tx: renal transplantation).

Compared with FGF-23 levels before transplantation, FGF-23 levels at the end of 3, 6, and 12 months were significantly decreased (Figure 1). At the same time periods, P as well as iPTH levels were also decreased. In addition, a significant increase was noted in Ca and 1,25(OH)_2_VitD levels. No significant changes were noted in 25(OH)VitD levels. TmPO_4_/GFR tended to increase, but the difference did not reach statistical significance. A statistically significant increase in TmPO_4_/GFR was noted between months 1 and 12 posttransplantation (2.3 ± 0.8 versus 3.3 ± 0.9, *P* = .04). The evolution of the aforementioned parameters over the first 12 months following kidney transplantation is shown in Table 1.

At 12 months posttransplantation all patients had normal FGF-23 levels (<50 pg/mL). 25(OH)VitD insufficiency (<30 ng/mL) was noted in 8 patients (44%) while 3 patients (17%) had 1,25(OH)_2_VitD deficiency (<20 pg/mL). iPTH levels >65 pg/mL were observed in 72% (*n* = 13) and 17% (*n* = 3) of the patients at the time of transplantation and at month 12, respectively. Patients with a history of parathyroidectomy had lower iPTH levels before transplantation and at month 12 posttransplantation compared to patients without parathyroidectomy (41 ± 40 versus 236 ± 172, *P* = .014 and 39 ± 19 versus 74 ± 50 pg/mL, *P* = .05, resp.). At 12 months posttransplantation, Ca levels were higher and P levels were lower in patients without parathyroidectomy (10.0 ± 0.3 versus 9.3 ± 0.4 mg/dL, *P* < .001 and 3.5 ± 0.6 versus 4.4 ± 0.6 mg/dL, *P* = .007, resp.). TmPO_4_/GFR was also lower in patients without parathyroidectomy (2.8 ± 0.6 versus 4.2 ± 0.9 mg/dL, *P* = .018) at 12 months posttransplantation. Patients receiving alfacalcidol tended to have lower iPTH values (48 ± 35 versus 93 ± 63 pg/mL, *P* = .113) at 12 months posttransplantation. FGF-23 levels also tended to be higher in patients receiving alfacalcidol (43.4 ± 19.8 versus 23.5 ± 5.8 pg/mL, *P* = .08, at 3 months posttransplantation).

**Figure 2:**
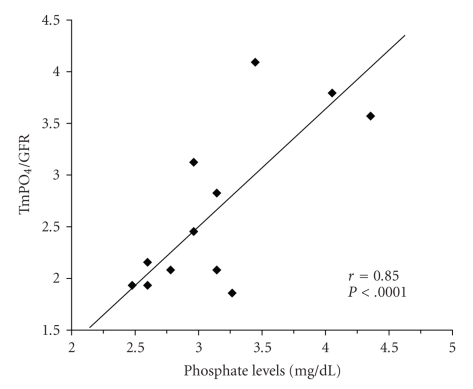
Correlation between TmPO_4_/GFR and P levels at 3 months postrenal transplantation.

A significant correlation was observed between TmPO_4_/GFR and P levels at 3, 6, and 12 months after transplantation (*P* < .0001, *P* < .002, and *P* < .0001, resp.) (Figure 2). In addition, pretransplantation FGF-23 levels were significantly correlated with P levels 3 months posttransplantation (*P* < .005). Pretransplantation FGF-23 levels were also correlated with TmPO_4_/GFR 3 months posttransplantation (*P* = .03). No correlations were observed between FGF-23 and vitamin D levels before or after renal transplantation.

Moderate hypophosphatemia (<2.5 mg/dL) was observed relatively infrequently after the first month posttransplantation. Up to 28% of kidney transplant recipients developed P levels <2.5 mg/dL transiently between 1 and 3 months posttransplantation. TmPO_4_/GFR was significantly lower in these patients (1.9 ± 0.1 versus 2.9 ± 0.8, *P* = .002). Posttransplantation 1,25(OH)_2_VitD levels were higher in hypophosphatemic patients (50.5 ± 10.7 versus 28.8 ± 13.3, *P* = .007). At month 12, only 1 patient (6%) still had hypophosphatemia. There was a tendency toward higher pretransplantation FGF-23 levels in patients that developed hypophosphatemia (451 ± 177 versus 302 ± 118 pg/mL, *P* = .147). Furthermore, patients that developed hypophosphatemia tended to have higher pre- and posttransplantation iPTH levels, but the difference did not reach statistical significance (280 ± 208 versus 135 ± 158 pg/mL and 76 ± 44 versus 47 ± 41 pg/mL, resp.).

Mean iPTH levels decreased by 64% at 3 months posttransplantation and remained stable thereafter. Pretransplantation iPTH correlated significantly with Ca and P levels 12 months posttransplantation (*P* = .044 and *P* = .031, resp.). During the first 6 months posttransplantation 3 patients (17%) temporarily developed Ca levels >10.4 mg/dL. At month 12 only 1 patient (6%) had mild hypercalcemia (Ca levels <12.5 mg/dL). Patients with posttransplantation hypercalcemia tended to have higher pretransplantation iPTH levels (298 ± 285 versus 151 ± 153 pg/mL, *P* = .205).

## 4. Discussion

The major findings of the present study are the following: (i) intact FGF-23 levels decrease dramatically after successful renal transplantation and remain within normal limits when graft function is good, (ii) TmPO_4_/GFR and P levels at month 3 correlate significantly with FGF-23 levels before transplantation, and (iii) no association between FGF-23 and 1,25(OH)_2_VitD levels was found.

In this prospective study, FGF-23 levels were determined in renal transplant recipients with stable renal function for a one year period. Intact FGF-23 was measured in order to avoid measurement of fragments that accumulate in end-stage renal disease and do not probably reflect endogenous FGF-23 production. In addition, FGF-23 levels before and after renal transplantation would be more comparable with the measurement of intact FGF-23 because in patients with good renal function fragment accumulation is not observed. We observed a significant reduction, already from the first trimester posttransplantation that reached 90% of pretransplant values. In the following measurements, at 6 and 12 months, there was a small further decline, and FGF-23 levels were within normal limits in all patients at month 12 posttransplantation. Phosphate levels and TmPO_4_/GFR at month 3 significantly correlated with FGF-23 levels before transplantation. Bones, and in particular osteoblasts, are considered the possible site of FGF-23 production [8]. In patients with chronic renal failure and secondary hyperparathyroidism that have undergone renal transplantation, it is expected that there is a greater production in the first trimester after transplantation, until osteoblasts become inactive and bone metabolism is suppressed. It has been reported that corticosteroids, calcineurin inhibitors, and mTOR inhibitors stimulate FGF-23 production [9, 10], even though in the first months after transplantation, with the use of higher doses of these drugs we as well as others observed the greater reduction [3–6]. Furthermore, hypophosphatemia which is the major stimulant for FGF-23 secretion is resolved. Phosphaturia in other solid organ transplantation is not observed, which points toward its relation either with secondary hyperparathyroidism or tubular damage. Intact FGF-23 contributes to the hypophosphatemia observed in the first month after transplantation, but after the first 6 months possible causes are persistent secondary hyperparathyroidism, tubular damage, and/or immunosuppressive drugs.

Our results are in agreement with those of Pande et al., who also observed FGF-23 level reduction as early as the fifth posttransplant day [5]. In the aforementioned study, not only FGF-23 was determined but also C-terminal fragments. For this reason, the excessive and rapid reduction could be due to increased urinary excretion. In the study by Evenepoel et al., the results also showed a significant reduction of FGF-23 levels. In this study, there are patients in the first trimester after transplantation with higher than normal intact FGF-23 levels but with lower glomerular filtration rate [6].

iPTH concentrations decrease progressively after renal transplantation. However, resolution of secondary hyperparathyroidism (SHPT) is incomplete 1 year after transplantation in about 50% of the recipients [11]. In our study, mean iPTH levels decreased by 64% at 3 months posttransplantation and remained low, but at the higher end of normal by month twelve. A significant correlation was observed between pretransplantation iPTH and Ca and P levels at 12 months posttransplantation, suggesting a role for SHPT in the development of postrenal transplantation hypercalcemia and hypophosphatemia. Patients that developed hypophosphatemia tended to have higher pre- and posttransplantation iPTH levels as well as higher pretransplantation FGF-23 levels. These findings suggest that FGF-23 and iPTH could act synergistically to cause phosphaturia, as has been previously suggested [4]. The role of iPTH in posttransplantation hypophosphatemia is further supported by the observation that renal transplant recipients that had undergone parathyroidectomy before transplantation had lower Ca and higher P levels 12 months posttransplantation. TmPO_4_/GFR was also higher in patients with a history of parathyroidectomy.

Hyposphosphatemia occurs early following renal transplantation and resolves almost completely at 1 year after transplantation [12]. Temporary mild hyposphosphatemia was observed in 28% of our patients at 3 months posttransplantation. In accordance with previous reports, only 1 patient (6%) remained hypophosphatemic at 12 months posttransplantation. Phosphate levels and TmPO_4_/GFR were strongly correlated indicating that low P levels after renal transplantation are the result of renal phosphate wasting. Pretransplantation but not posttransplantation FGF-23 levels correlated with P levels as well as TmPO_4_/GFR at 3 months posttransplantation. This finding is consistent with previous studies that implicate FGF-23 in postrenal transplantation hyposphosphatemia [3–6].

Up to 91% of our patients had sufficient 1,25(OH)_2_VitD levels between 3 and 12 months after renal transplantation. No association was found between FGF-23 and 1,25(OH)_2_VitD levels.

 Our study has some advantages. One is that FGF-23 levels were determined with an assay that does not detect C-terminal fragments. Another is that serial measurements of FGF-23 after renal transplantation were conducted. Possible limitations, on the other hand, are the small number of patients and the use of activated vitamin D analogs and calcium salts, which may have confounded our results.

In conclusion, FGF-23 levels decrease dramatically after successful renal transplantation and remain within normal limits when graft function is good. iPTH and P levels also decrease significantly after renal transplantation, while Ca and 1,25(OH)_2_VitD increase. Pretransplantation FGF-23 levels correlate with TmPO_4_/GFR and P levels 3 months posttransplantation. Pretransplantation iPTH also correlate with Ca and P levels 12 months posttransplantation. No association was found in this study between FGF-23 and 1,25(OH)_2_VitD levels. Further studies are needed to elucidate the role of FGF-23 in P and 1,25(OH)_2_VitD metabolism after renal transplantation.

## Figures and Tables

**Table 1:** Evolution of studied parameters over the first 12 months following kidney transplantation.

	pre-Tx	3 months	6 months	12 months	*P* ^§^
Phosphate (mg/dL)	5.7 ± 1.7	3.1 ± 0.7	3.2 ± 0.8	3.8 ± 0.7	< .005
(2.5–4.5)^#^					
Calcium (mg/dL)	9.2 ± 0.9	9.8 ± 0.5	9.9 ± 0.6	9.9 ± 0.6	< .01
(8.8–10.4)^#^					
25(OH)VitD (ng/mL)	37.5 ± 23.4	29.9 ± 10.9	34.1 ± 14.2	34.8 ± 15.9	NS
(7.6–75)^#^					
1,25(OH)_2_VitD (pg/mL)	18.5 ± 6.7	34.6 ± 14.3	39.2 ± 12.3	34.7 ± 15.4	< .05
(29.6–65.1)^#^					
iPTH (pg/mL)	181 ± 171	67 ± 41	70 ± 40	64 ± 45	< .05
(10–65)^#^					
FGF-23 (pg/mL)	346 ± 146	37 ± 19	32 ± 20	31 ± 14	< .01
(8.2 –54.3)^#$^					
TmPO_4_/GFR		2.6 ± 0.9	2.9 ± 1.0	3.3 ± 0.9	NS^& ^
(2.5–4.2)^#^					
ALP (IU/L)	110 ± 78	81 ± 28	75 ± 28	79 ± 33	< .05
(30–120)^#^					
Serum creatinine (mg/dL)	7.6 ± 3.1	1.3 ± 0.2	1.3 ± 0.3	1.3 ± 0.2	< .001
(0.66–1.1)^#^					

Values are expressed as means ± SD.

^§^Paired *t*-test between pre-Tx and post-Tx values.

^&^Paired *t*-test between 3 and 12 months post-Tx.

^#^Normal values.

^$^Yamashita H et al. European Journal of Endocrinology, 2004.
